# Effect of Clinical, Biochemical, and Genetic Variations on Medical Management in Filarial Chyluria: A Prospective Study at a Tertiary Care Centre in North India

**DOI:** 10.7759/cureus.17292

**Published:** 2021-08-18

**Authors:** Shriya Pant, Apul Goel, Pravin K Gangwar, Akancha Pandey, Jyotsna Agarwal, Prashant Gupta

**Affiliations:** 1 Urology, King George's Medical University, Lucknow, IND; 2 Gynecology, King George's Medical University, Lucknow, IND; 3 Microbiology, Dr. Ram Manohar Lohia Institute of Medical Sciences, Lucknow, IND; 4 Mircrobiology, King George's Medical University, Lucknow, IND

**Keywords:** microfilariae, hematochyluria, filariasis, proteinuria, chyluria, triglycerides, cholesterol, milky urine, mannose-binding lectin, infectious disease

## Abstract

Objective

To analyze the effect of clinical, biochemical factors, and Mannose Binding Lectin 2 (*MBL2*) gene variations on medical management in filarial chyluria (FC) patients.

Material and methods

We conducted a study between March 2013 and April 2016. *MBL2*polymorphisms were genotyped in confirmed 101 medically-treated cases of FC. Demographic, clinical, and biochemical variables were compared between remission and failure groups. Genotyping of *MBL2* codon 54 and promoter -221 were undertaken by polymerase chain reaction. Genotype frequencies were compared with clinical and biochemical variables and medical treatment outcomes (remission/failure). The association between genotypes and treatment response was estimated by OR and 95% CI and generated by the chi-square test.

Results

The mean age was 36.9±10.28-years and the male-female ratio was 3:1.2. Sixty-six patients had remission (Group-A) while 35 had recurrence (Group-B) at a mean follow-up of 21 months. The success rate for medical therapy was 65.35%. There was no statistical difference observed in the demographic profile of the two groups. On multivariate analysis, patients in Group-B had a higher grade of chyluria (p=0.005), had experienced greater number of disease attacks in the past (p=0.022), and had higher urinary triglyceride levels (TG) (p<0.001) as compared to Group-A patients. A significant association of *MBL2* codon 54 genotypes was observed with the recurrent presentation of chyluria (p=0.044), grade of chyluria (p=0.028), and urinary TGs (p=0.001). However, genotype distribution at -221 did not show association with clinical and biochemical parameters of FC patients. The distribution of genotypes at codon 54 differed significantly between remission and failure/recurrence group; the variant genotype BB was significantly higher in the recurrence or failure group (OR:6.00; 95%CI, 1.00-35.91; p=0.050). However, frequencies of variant genotype YX and recessive group YX+XX of *MBL2* -221 promoter was higher in remission group (OR:2.97;95%CI, 1.23-7.13; p=0.018 and OR:2.76; 95%CI, 1.80-6.50; p=0.020), respectively, showing that genetic variant may be associated with response to medical therapy.

Conclusion

Higher grade of chyluria, a higher number of disease attacks in the past, and higher urinary TGs levels were clinical predictors of poor response to medical treatment. Our results showed that the variants of *MBL2* genes have an impact on treatment outcomes in FC patients. These observations may be limited by sample size.

## Introduction

Chyluria is caused by a rupture of lymphatic varices into the urinary excretory system. It is frequently episodic, with episodes lasting for days or weeks. The onset may be insidious or sudden. Chyluria tends to be more pronounced in the morning or after a fatty meal. Prolonged chyluria may result in weight loss leading to, hypoproteinaemia, lymphopenia, and anemia [1]. Chyluria is endemic in India, Bangladesh, Japan, Indonesia, China, Taiwan, parts of Africa, Australia, and South America. Worldwide, the most common cause of parasitic chyluria is lymphatic filariasis [2]. *Wuchereria bancrofti* infestation is responsible for >95% of parasitic chyluria in India [3]. In endemic areas, approximately 10% of the population are infected, of which 10% eventually develop chyluria [4]. About one-third of all the infected people in the world live in India. It is recognized as a tropical disease and is associated with spontaneous remissions that occur in >50% of cases. A low-fat diet, bed rest, and high fluid intake reduce the risk of urinary stasis and clot formation. Chyluria can be classified into mild, moderate, or severe [5]. Although the cause of filarial chyluria (FC) is well-known, the reason for susceptibility is unknown. In an effort to identify the reason for susceptibility for FC, we examined the effect of *MBL2* gene polymorphisms on the progression of FC infection in the North Indian population [6]. Mannose-binding lectin (MBL) is a C-type lectin with a collagen-like domain-1 that plays an important role in the innate immune defense against disease. Variations at the amino acid coding region may lead to the structural changes in MBL protein, causes a significant reduction in the MBL serum levels, In addition, variations at promoter regions may affect transcript levels of MBL2 protein that ultimately translate to lesser protein expression. Variations in *MBL2* gene are responsible for poor opsonization and are associated with increased susceptibility to various infections, including filarial infection [7-9]. We confirmed in our previous publication that there is a possible association between *MBL2* genotypes with FC infection [6].

FC is characterized by an unpredictable response to treatment with some patients responding well while others showing poor response. The reason for this is also unknown. Efforts have been made to predict the response to medical therapy using various clinical and biochemical parameters [10]. However, to the best of our knowledge, an association between genetic variations and treatment response in patients with FC has not been investigated yet. This study aimed to determine whether genetic variants in the *MBL2* gene influence treatment response in FC patients. Knowledge about the genetic influence on this disease and the predictors of response will not only improve treatment decisions but also help in understanding the basis of medical response to treatment

## Materials and methods

Study design and subjects

This study was approved by the Institutional Ethics Committee, King George's Medical University, Uttar Pradesh, India (Reference code number: 5534/R.cell-13), and a written informed consent form was taken from all patients, using a standardized questionnaire. We enrolled 108 confirmed FC patients with age >18-years. Filarial etiology was confirmed by using Giemsa-stained thick and thin smear examination followed by diethylcarbamazine (DEC) provocation test [8,11], immunochromatographic card test (ICT) of BinaxNOW® Filariasis (Abbott Laboratories, IL, United States) [12] and IgG/IgM-combo rapid antibody test (CTK Biotech Inc., CA, USA) [13]. Patients found positive by any of these tests were confirmed to have FC. The patients were enrolled from March 2013 to April 2016 (including patient recruitment between March 2013 to April 2016 and follow-up till November 2017) at a tertiary care hospital located in a filariasis endemic zone. Patients below 18 years of age and/or with non-parasitic chyluria, malignancy, non-chyluria whitish-cloudy urine [3], renal failure, pregnancy, and uncontrolled diabetes were excluded. Patients had full freedom to deny participation in the study or to opt out anytime during the course of study. Seven patients were excluded due to various reasons including withdrawal by the patient, loss to follow-up, and negative diagnostics tests (non-parasitic chyluria). Data of the patients were added to an organized sheet based on demographic features (age, sex, and location), and complete clinical investigations of the disease (duration, number of previous episodes, duration of current episodes, grade of chyluria, treatment taken, and response to treatment). Urinary investigations included routine urinalysis, culture, presence of chyle (gross assessment, ether dissolution test followed by microscopy), quantitative analysis of urinary TGs, and cholesterol levels using the biochemical auto-analyzer.

The severity of chyluria was assessed with a widely used clinical grading system [14,15] incorporating minor modifications based on the severity of hematuria as described below:

Grade-I: Single episode or ≤ 1-episode/year of hematuria of < 24 h duration, not associated with the passage of blood clots or anemia

Grade-II: Single episode or ≤ 1-episode/year of hematuria of ≥ 24 h duration, not associated with the passage of blood clots or anemia

Grade-III: ≥2 episodes/year or hematuria associated with the passage of blood clots or anemia.

Anti-filarial treatments, supportive medicines such as hematinics (if needed), rest, and dietary changes were initially used to treat the patients. The anti-filarial treatment regimen includes one to two courses of DEC in a dose of 6mg/kg/day (three divided doses) for three weeks, doxycycline 100mg twice a day for three weeks, a single dose of albendazole and ivermectin with a 15-day gap between the two courses. High-protein diet, green leafy vegetables, reduced fat intake (25-50g/day) with the inclusion of lipids containing medium-chain triglycerides, and high fluid intake were all prescribed as dietary adjustments. All patients were provided a diet plan with general dietary modification suggestions. However, no explicit suggestions for specific amounts or food types were given. After collecting the above details, 5-ml peripheral blood was obtained from each subject to perform diagnostic tests and molecular analysis as described previously.

Genotyping

Genomic DNA was extracted from whole blood using a commercially available DNA isolation kit, Quick-gDNA™ MiniPrep (Zymo Research Corporation, CA, USA) as per the manufacturer’s protocol. 100-ng of DNA was used as the template for subsequent polymerase chain reaction (PCR). Genotyping of *MBL2* gene at site codon54: rs1800450 and -221:rs7096206 was done by PCR with specific primer pairs as described in our previous study [6].

Follow-up protocol

Patients were initially followed at one month, followed by a three-month visit. They were then advised to visit the clinic in case of relapse or problem. Those patients who could not attend the clinic for follow-up were enquired telephonically regarding recurrence. Successful treatment was defined as a complete resolution of symptoms with stable remission until the last follow-up. Recurrence/failure was defined as patients who did not respond to a prescribed regimen of two courses of drug combinations, as well as suitable dietary and supportive measures, or who presented with repeated symptoms after an initial response. Genotype frequencies of FC patients were compared with demographic, clinical, biochemical parameters, and outcomes (remission/recurrence) of medical therapy.

Statistical analysis

All the data analyses were carried out using SPSS Statistics for Windows, Version 17.0, released 2008 (SPSS Inc.. Chicago, US). The categorical data were summarized as number and percentage and continuous data in mean±standard deviation (SD). Continuous and discrete variables were compared using the chi-square test (χ2) and Kruskal-Wallis test, wherever applicable. Statistical analysis for genotypes was done with a statistical online calculator available at the VassarStats website for statistical computation. Genotype data were compared between remission (Group A) and recurrence/failure (Group-B) by using chi-square and Fisher exact probability tests. Wherever significant differences were observed, further analyses were done to calculate OR and the corresponding 95% CI. The p-value and 95% CI were used to assess the strength of association.

## Results

Of the 101 patients, 66 (61.11%) experienced and continued to be in remission of chyluria at a mean follow-up period of 21 months (range: 12-26) (Group-A). Nine patients failed to respond to medical treatment and were labeled as treatment failures while 26 patients experienced a recurrence of symptoms after an initial response. These two categories of patients were clubbed into Group-B. The mean age of Group-A (remission) and Group-B (recurrence/failure) patients was 37.91 years (SD 9.27) and 35.0 years (SD 11.86), respectively. Group-A patients were compared with Group -B to analyze the predictors of response to medical therapy (Table 1). There were no statistical differences in demographic distribution between remission and recurrence (treatment failure) groups. The patients in Group-B had a significantly higher grade of disease (p=0.005), had experienced a greater number of recurrent infections (p=0.004), had a longer duration of the disease (p=0.031), and higher number of previous attacks (p<0.001). The differences in biochemical parameters between Group-A versus Group-B are shown in Table 1. Patients in Group-B had a significantly elevated level of urinary TGs (p<0.001) and cholesterol loss (p=0.038) compared to patients in Group-A.

**Table 1:** Demographic, clinical, and biochemical profile as predictor of response to medical therapy in filarial chyluria patients

Variables	Remission (Group A) (n=66) (%)	Failure (Group B) (n=35) (%)	Unadjusted OR (95% CI)	p-value
Age (years)	37.91± 9.27	35.0±11.86	0.97 (0.93-1.01)	0.177
Sex: female male	15 (22.7) 51 (77.3)	14 (40.0) 21 (60.0)	Ref. 0.44 (0.18-1.07	0.071
Residence: rural urban	45 (68.2) 21 (31.8)	25 (71.4) 10 (28.6)	Ref. 0.86 (0.35-2.10)	0.736
Type of presentation: primary recurrent	41 (62.1) 25 (37.9)	11 (31.4) 24 (68.6)	Ref. 3.57 (1.50-8.54)	0.004
Grade of chyluria: I II III	29 (43.9) 35 (53.0) 2 (3.0)	10 (28.6) 17 (48.6) 8 (22.9)	Ref. 1.41 (0.56-3.55) 11.60 (2.10-64.01)	0.467 0.005
Duration of chyluria (month): mean± SD	20.29±36.69	52.51±87.85	1.01 (1.00-1.02)	0.031
Number of previous episodes: mean± SD	1.24±1.22	2.34±1.25	1.97 (1.38-2.83)	<0.001
Urine triglycerides (g/dl): mean± SD	161.60±127.58	356.89±201.13	1.01 (1.00-1.01)	<0.001
Urine cholesterol (g/dl): mean± SD	16.06±17.22	25.34±16.49	1.04 (1.00-1.07)	0.038

A multivariate logistic regression analysis (remission versus recurrence) with factors affecting the response to medical therapy, is shown in Table 2. FC patients with a high grade of disease (p=0.05), greater number of previous episodes (p=0.002), and higher urinary TGs levels (p=<0.001) had a high probability of disease recurrence, which are the predictors of poor response to medical management.

**Table 2:** Multivariate logistic regression analysis of factors affecting the outcome of medical therapy (remission vs recurrence/failure)

Variables	Beta coefficient	Adjusted OR (95% CI)	p-value
Type of presentation: primary recurrent	-1.215	Ref. 0.30 (0.04-2.15)	0.229
Grade of chyluria: I II III	0.029 2.193	Ref. 1.03 (0.28-3.77) 8.97 (0.89-90.50)	0.965 0.05
Duration of chyluria (month)	0.004	1.00 (0.99-1.02)	0.496
Number of previous episodes	0.890	2.43 (1.14-5.20)	0.022
Urine triglycerides (g/dl)	0.008	1.01(1.00-1.01)	<0.001
Urine cholesterol (g/dl)	0.020	1.02 (0.99-1.05)	0.0180

The distribution of* MBL2* codon 54 genotype frequencies (wild (AA)/ heterozygous (AB)/ mutant (BB)) with clinical and biochemical parameters of FC patients is summarized in Table 3. A significant association of *MBL2* codon 54 genotypes was observed with the recurrent presentation of chyluria (p=0.044), grade of disease (p=0.028), and urinary triglycerides (p=0.001).

**Table 3:** The association between MBL2 codon 54 rs1800450 and promoter -221 rs7096206 genotypes and the clinical and biochemical features of filarial chyluria patients MBL2: Mannose binding lectin 2

Variables	Wild (AA) (n=64) (%)	Hetero (AB) (n=31) (%)	Mutant (BB) (n=6) (%)	p-value	Wild (YY) (n=51) (%)	Hetero (YX) (n=44) (%)	Mutant (XX) (n=6) (%)	p-value
Type of presentation: primary recurrent	39 (60.9) 25 (39.1)	11 (35.5) 20 (64.5)	2 (33.3) 4 (66.7)	0.044	30 (58.8) 21 (41.2)	18 (40.9) 26 (59.1)	4 (66.7) 2 (33.3)	0.163
Grade of chyluria: I II III	29 (45.3) 33 (51.6) 2 (3.1)	9 (29.0) 15 (48.4) 7 (22.6)	1 (16.7) 4 (66.7) 1 (16.7)`	0.028	21 (41.2) 27 (52.9) 03 (5.9)	16 (36.4) 21 (47.7) 7 (15.9)	2 (33.3) 4 (66.7) 0 (0.0)`	0.459
Duration of chyluria (months): mean± SD	31.6±65.94	32.61±14.0	23.50±18.50	0.17	32.40±63.86	32.16±61.90	18.30±26.72	0.38
Number of previous episodes: mean± SD	1.42±1.27	2.0 ± 2.0	1.83±2.50	0.17	1.43±1.25	1.82±1.40	1.80±1.47	0.39
Urine triglycerides (g/dl): mean± SD	196.4±4	279.60±218.0	319.70±279.8	0.001	206.0±181.30	250.49±170.60	271.20±265.70	0.23
Urine cholesterol (g/dl): mean± SD	18.2±16.86	21.29±19.1	20.41±22.0	0.28	21.80±23.0	16.48±8.20	18.26±8.94	0.85

We evaluated the effect of *MBL2* codon 54 variations on treatment response (Table 4). The frequency of wild genotype AA was 75.0% in Group-A and 25% in Group-B. By using AA genotype as a reference group, the AB genotype, the BB genotype, and the recessive model AB+BB were found significantly associated with response to medical treatment (OR:2.81; 95%CI, 1.14-6.94; p=0.034, OR:6.00; 95%CI, 1.00-35.91; p=0.050, and OR:3.17, 95%CI, 1.34-7.47; p=0.009). The findings suggest that the frequency of variant genotype BB was two-fold higher in Group-B patients, 66.7%, compared to Group-A, which was 33.3%.

We next examined the association between *MBL2* -221 (wild (XX)/ heterozygous (XY)/ mutant (YY)) genotypes and clinical, biochemical profile of FC patients. No significant association was observed as seen in Table 3.

In addition, to determine the potential effect of *MBL2* -221 variation on the outcome of medical treatment, we genotyped this variant with Group-A and Group-B patients. We found that the frequency of variant genotype YX and recessive group YX+XX of promoter *MBL2* -221was significantly higher in Group-A (OR:2.97; 95%CI, 1.23-7.13; p=0.018), (OR:2.76; 95%CI, 1.80-6.50; p=0.020), respectively. This showed a significant association between genotypes and treatment response (Table 4).

**Table 4:** Association between MBL2 codon 54 rs1800450 and promoter -221 rs7096206 genotypes and outcome of medical therapy MBL2: Mannose binding lectin 2

*MBL2* gene	Medical therapy (n=101)				
	n =101 (%)	Remission (Group A) (n=66)	Recurrence/failure (Group B) (n=35)	OR (95% CI)	p-value
Codon 54 (rs1800450) Wild (AA) Hetero (AB) Mutant (BB) AB+BB	64 31 6 37	48 (75) 16 (51.6) 2 (33.3) 18	16 (25) 15 (48.4) 4 (66.7) 19	Ref. 2.81 (1.14-6.94) 6.00 (1.00-35.91) 3.17 (1.34-7.47)	0.034 0.050 0.009
-221 (rs7096206 ) Wild (YY) Hetero (YX) Mutant (XX) YX+XX	51 44 6 50	39 (76.5) 23 (52.3) 4 (66.7) 27	12 (23.5) 21 (47.7) 2 (33.3) 23	Ref. 2.97 (1.23-7.13) 1.63 (0.26-9.99) 2.76 (1.80-6.50)	0.018 0.630 0.020

## Discussion

The last few years have established the importance of single nucleotide polymorphisms (SNPs) as the most common and effective type of genetic diversity studied in relation to susceptibility to various infections, and they can be considered as markers of many severe diseases [16]. Variation in susceptibility to infection is predisposed by genetic factors of the host and the first line of protective immunity is innate immunity [17]. Thus, genetic differences in components of innate immunity may lead to susceptibility to infection. In our previous study, we evaluated the association of the *MBL2* gene polymorphism with FC infection in the North Indian population. The purpose of this study is to see the impact of the* MBL2* variant on clinical and biochemical parameters and response to outcome of medical treatment.

The reported success rates for medical treatment of FC have been highly variable and high relapse rates have been suggested after successful initial treatment. We previously reported a success rate of 70.3% to medical therapy in a study population [10]. Ohyama et al. observed a success rate of medical treatment being 61% [18]. Our study with a different patient population showed comparable results with an overall success rate of 65.35% [19]. The study of Tandon et al. also reported comparable outcomes with a cure rate of 62% [20]. 

In this study, we tried to identify the clinical predictors of recurrence of FC and also analyze the impact of demographic, clinical, and biochemical factors as well as *MBL2* genotypes on response to medical treatment. There was no statistical difference in the demography of the two groups of patients. Patients in Group-B had a significantly higher grade of disease (p=0.005), had a recurrent presentation of chyluria (p=0.004), duration of chyluria was longer (p=0.031), had experienced a greater number of disease attacks in the past (p<0.001), and showed poor response to medical therapy. In a comparison of urinary lipids, patients with treatment failure (Group-B) had significantly higher urinary TGs (p<0.001) and urinary cholesterol loss (p<0.038) as compared to remission patients Group-A. The triglycerides and cholesterol that are lost in urine come from intestinal chylomicrons. Similar to our previous observations, we noticed that the patients in Group-B had significantly higher urinary TGs and cholesterol loss at baseline compared with Group-A patients [8]. So, urinary triglycerides and cholesterol loss at baseline is a better predictor of response to medical therapy.

In this study, we analyzed the association of *MBL2* codon 54 genotypes (wild (AA), heterozygous (AB), and mutant (BB)) with the clinical and biochemical profile of medically-treated patients. Our results indicate that the patients with mutant genotype (BB) had a significantly higher grade of disease (p=0.028) and a higher level of urinary TGs (p=0.001). Likewise, in another study, it has been reported that the variant genotype is associated with disease progression [21]. Similarly, the frequency of AB/BB genotypes was significantly higher in patients with recurrent chyluria (p=0.044). The frequency of wild genotype AA was 75.0% in Group-A (remission) and 25% in Group-B (recurrence/failure). This study suggests that patients with AA genotype are more likely to be cured by medical therapy. Mutant genotype (BB) and recessive model AB+BB of *MBL2* codon 54 gene were found to be significantly associated with poor response (failure) to medical treatment (OR:2.81; 95%CI, 1.14-6.94; p=0.034, OR:6.00; 95%CI 1.00-35.91; p=0.050 and OR:3.17, 95%CI, 1.34-7.47; p=0.009).

On the other hand, no association was observed between *MBL2* -221 genotypes (wild (XX)/ heterozygous (XY)/ mutant (YY)) and the clinical and biochemical profile of patients. On comparing the genotypic data with treatment outcome, we observed a significant association of *MBL2* -221 genotypes with treatment response.

A few limitations of the present study need to be considered. First, this is a preliminary prospective study with a relatively small number of patients in inclusion and exclusion criteria. Second, the follow-up time is short. There are different manifestations of lymphatic filariasis like elephantiasis of the penis and scrotum and hydrocele. In this study, we selected only FC patients, because urologists (the study was conducted in the Department of Urology) primarily deal with chyluria patients. Other common chronic manifestations are also sometimes addressed with the help of a urologist; however, in our center, it is mainly managed by plastic surgeons and general surgeons. We, therefore, restricted our study population to chyluria. There are limitations in the serological tests used for the diagnosis of filarial etiology. Despite these limitations, these data provide insights into the relationship between *MBL2* gene polymorphism and treatment response in FC patients and could form a basis for further studies in a large-scale population.

## Conclusions

Our study suggested that patients with a higher clinical grade, a higher number of previous attacks, and high urinary TG and cholesterol have a poorer outcome to medical therapy with a success rate of 65.5%. In this preliminary study, homozygous wild genotypes of *MBL2* codon 54 and *MBL2* -221 seemed to be linked to better response while mutant genotype was associated with poor response to medical therapy. Additionally, *MBL2* codon 54 genotypes seem also to be associated with clinical and biochemical parameters of FC patients. We conclude thatthe* MBL2* codon 54 gene may be a good candidate for future studies, provided evidence that these SNPs may be suggested as a clinical predictor in treating patients of FC. To the best of our knowledge, this is the first study from North India that has tried to explore the genetic variants of *MBL2* genes in FC patients along with treatment responses. Therefore, we may further suggest systematic analyses of the genetic variants/mutations of *MBL2* genes in different ethnic groups, regions, and expanded sample size to confirm our findings and understand the association of gene polymorphism with clinical and treatment response in the larger context.
